# Is It Time We Better Understood the Tests We are Using for Return to Sport Decision Making Following ACL Reconstruction? A Critical Review of the Hop Tests

**DOI:** 10.1007/s40279-019-01221-7

**Published:** 2019-11-19

**Authors:** William T. Davies, Gregory D. Myer, Paul J. Read

**Affiliations:** 1 Aspetar, Orthopaedic and Sports Medicine Hospital, Sports City Street, P.O. Box 29222, Doha, Qatar; 2 Division of Sports Medicine, The SPORT Center, Cincinnati Children’s Hospital Medical Center, Cincinnati, OH USA; 3 Departments of Pediatrics and Orthopaedic Surgery, College of Medicine, University of Cincinnati, Cincinnati, OH USA; 4 The Micheli Center for Sports Injury Prevention, Waltham, MA USA

## Abstract

There has been a move towards a criterion-based return to play in recent years, with 4 single-leg hop tests commonly used to assess functional performance. Despite their widespread integration, research indicates that relationships between ‘passing’ ‘hop test criteria and successful outcomes following rehabilitation are equivocal, and, therefore, require further investigation. This critical review includes key information to examine the evolution of these tests, their reliability, relationships with other constructs, and sensitivity to change over time. Recommendations for how measurement and administration of the tests can be improved are also discussed. The evidence presented in this review shows that hop tests display good reliability and are sensitive to change over time. However, the use of more than 2 hop tests does not appear to be necessary due to high collinearity and no greater sensitivity to detect abnormality. The inclusion of other hop tests in different planes may give greater information about the current function of the knee, particularly when measured over time using both relative and absolute measures of performance. It is recommended that the contralateral limb be tested prior to surgery for a more relevant benchmark for performance, and clinicians are strongly advised to measure movement quality, as hop distance alone appears to overestimate the recovery of the knee.

## Key Points

**Table:** 

While the ACL hop tests display adequate reliability, the current evidence indicates a lack of consistency in their capacity to predict successful outcomes following rehabilitation, either in terms of returning to previous performance levels, or identifying those at a greater risk of re-injury.
The current practice of using 4 hop tests to inform decision making appears to be unnecessary. Using fewer horizontal hop tests provides clinicians with an opportunity to examine a wider range of physical constructs that may offer broader insights into the athlete’s readiness to return to sport.
Hop distance/time should not be the sole measure or performance, and other factors relating to movement control should be assessed and form part of the RTS decision-making process. In addition, measuring the trajectory of progress over time may also give the clinician more useful information for decision making.

## Introduction

Return to sport (RTS) decision making following anterior cruciate ligament reconstruction (ACLr) is a complex process [1]. It has been indicated that between 65 and 79% of elite athletes return to their prior injury level of competition [2–4]; however, 12 and 23% of elite athletes have been shown to either reduce their playing level or end their careers, respectively, within a 3-year follow-up period [4]. In addition, there is a relatively high risk of re-injury, whereby up to 20–25% of athletes will experience a contralateral tear or graft re-rupture [5, 6]. A high prevalence of chronic knee pain and functional limitations has also been reported in athletes following ACLr [7].

**Fig. 1:**
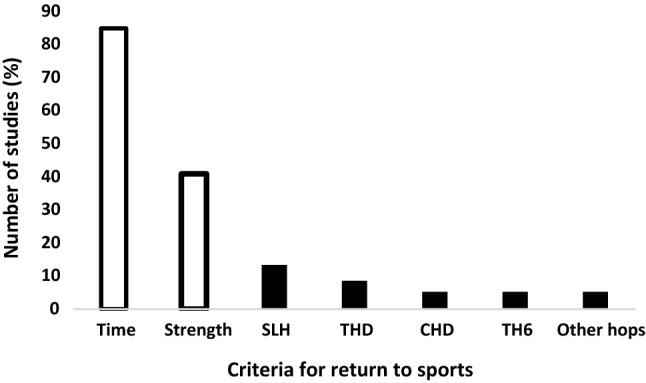
Criteria most used as part of the return to sport decision (data extracted from Burgi et al. [8]). *SHD* single hop for distance, *THD* triple hop for distance, *CHD* cross-over hop for distance, *6TH* 6 m timed hop

Within the available literature, RTS has been defined using varied terminology [3, 4, 8], making comparisons of the findings across different studies challenging. RTS should be viewed as a continuum paralleled with recovery and rehabilitation inclusive of return to participation, followed by competitive sport, with the aim of returning to performance [9]. Thus, generally, RTS is most commonly defined as a return to unrestricted training or competition [3, 4, 8]. Traditionally, the time frame from reconstruction has been used as the main criteria to establish whether an athlete is ready to RTS (Fig. 1) [10–12]. An average time frame of approximately 7 months has been reported [13], although accelerated rehabilitation programs have been advocated which target RTS before 6 months [13, 14]. More recent evidence indicates that re-injury rates can be reduced by 50% for every month return to sport is delayed up to 9 months, with no further reductions in risk shown after this point [15]. Some evidence suggests that time frames of up to 2 years may even be necessary to moderate risk of re-rupture [16, 17]. These inconsistencies in the current literature concerning when an athlete should RTS indicate that the use of temporal guidelines alone to measure readiness to RTS should be avoided.

In light of the fact that no associations have been observed between time from surgery and functional deficits in athletes who are in the process of completing their RTS [18, 19], a progressive shift towards a more criterion-based progression has ensued. A range of tests are utilized in various combinations, including isokinetic strength assessment of the quadriceps and hamstrings, single leg hop testing and other functional movement patterns (Fig. 1) [10, 11, 20, 21]. Available evidence indicates that passing a battery of assessments for RTS, including strength and hop tests, reduces the risk of re-injury [15, 22]. However, a recent report indicates patients who returned earlier because of passing designated criteria had an increased risk of contralateral ACL injury but they also displayed superior knee function and higher activity levels at the mid-term (4-year) follow-up [23]. When interpreting these data, it should be considered that those with better function normally return to a higher level of competition and have greater exposure which was not controlled for in this study. A more recent meta-analysis has also questioned the validity of current RTS test protocols, reporting no associations between re-injury [20] and even greater risk on the contralateral limb [21]. However, these findings have been challenged recently [24], with the authors questioning the validity of pooling studies with substantial methodological and clinical heterogeneity. Thus, it is prudent to more closely examine the constructs of existing RTS assessments.

**Fig. 2:**
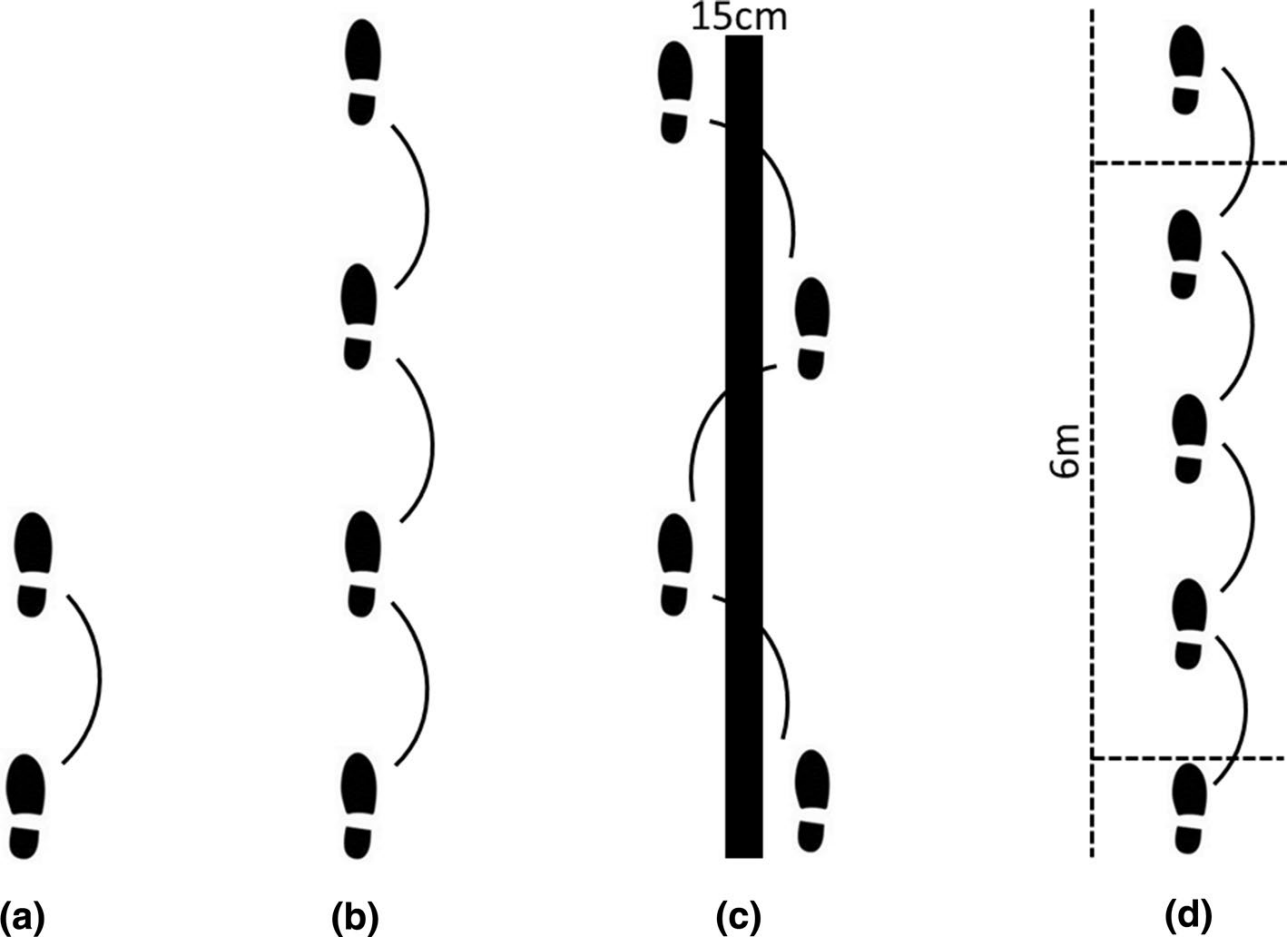
Depiction of the 4 single leg hop tests commonly used in return to sport protocols: **a** single hop for distance, **b** triple hop for distance, **c** cross-over hop for distance, **d** 6-m timed hop

A battery of single leg hop tests (Fig. 2) has most frequently been used within the available literature to assess ‘functional’ performance and has become the staple (in addition to isokinetic testing) of an ACLr RTS test battery [3, 15, 22, 25–27]. The adoption of these tests is likely in part due to their practical utility and ease of administration. Objective decisions can be made by directly comparing the reconstructed to uninvolved leg, creating a limb symmetry index (LSI). Scores greater than 90% LSI have been suggested as a clinical criterion to ‘pass’ and subsequently complete rehabilitation [28, 29]. A recent study showed that at 6 months, each of the 4 hop tests could predict return to previous levels of sport at 2 year post-surgery [30] and that patients with single hop for distance (SHD) and triple hop for distance (THD) scores greater than 85% LSI at time of RTS were more likely to return to their previous levels [31]. Specifically, the 6 m timed hop (T6H) and SHD have been shown to be the strongest predictors of those who are more likely to RTS [30–32] and THD scores (relative to body height and LSI) displayed the strongest predictive ability for re-injury [33].

In spite of the widespread integration of ‘hop testing’, recent literature has shown that athlete performance during these tests at 6 month post-surgery was unable to predict return to play at 12 months after rehabilitation, whereas strength testing and the subjective patient rating of function provided more relevant information [3, 25]. In addition, single leg hop for distance test scores have not been able to differentiate athletes who had RTS 2 years following reconstruction versus those who had not [34]. Similarly, passing a battery of hop tests has been associated with lower re-rupture rates; however, in these studies, only isokinetic strength measures showed direct associations with ACL re-injury rate [15, 22]. Cumulatively, these data indicate that a critical examination of ‘hop tests’ is warranted to determine their suitability to identify functional deficits following ACLr.

### The Evolution of Hop Testing as Measurement Tool to Assess Function Following ACLr

Hop testing was first cited in the early 1980s with a number of papers espousing their use to evaluate closed chain performance in athletes with ACL injury [35, 36]. These studies utilized the SHD to quantify function and concluded the test may be useful in guiding RTS [36]. The remaining 3 hop tests first appeared in the literature during the early 1990s, with the introduction of the T6H, followed by the cross-over hop for distance (CHD) and the THD [37, 38]. These studies were among the first to objectively assess functional performance as part of a criteria driven RTS process. A limb symmetry index ratio (sum of the involved leg/uninvolved leg × 100) was proposed to assess the likelihood of a ‘functional abnormality’ in the ACL reconstructed knee. Data from a non-injured population indicated that 81% of people displayed symmetry greater than 90%; 93% of the cohort-achieved symmetry greater than 85%, and all had LSI values greater than 80% [37]. As a result, target guidelines for injured athletes to achieve were set at 85%, with lower values considered as abnormal symmetry [37, 39]. Shortly after, a study reporting differences in LSI between athletes who returned to high-level sports, and those who failed rehabilitation demonstrated that successful subjects averaged over 90% LSI in their functional hop tests, whereas those who failed (6 subjects) had scores lower than 90% [28]. The requirement for a higher LSI threshold was also shown in a more recent study, with 100% of a ‘normal’ uninjured population demonstrating symmetry values greater than 90% for all 4 hop tests [29]. These studies have helped to shape current guidelines, providing an objective measure for use in evaluating performance during RTS testing [15, 22].

## Examination of the Hop Tests (Measurement Error, Performance Constructs and Temporal Evaluation)

### Reliability

Early research has shown the three hops for distance tests to demonstrate excellent rank-order repeatability (ICC > 0.95), with the authors stating that due to a low standard error mean, any discrepancy within the measures would occur within an acceptably small range [40]. Strong reliability of the LSI has also been indicated, with ICC values of 0.92, 0.88, and 0.84 reported for single, triple, and cross-over hop for distance, respectively [41]. The T6H has consistently reported lower values (ICC = 0.66 and 0.82) [40, 41]. This in part may be due to the protocol frequently adopted within the available literature which has used a stopwatch for measurement. These protocols can demonstrate good reliability; however, there is a trend of systematic bias and faster times when compared to automatic timers [42, 43]. In addition, larger absolute errors are present [44] compared to automatic timing devices (photocells etc.). Furthermore, more bias is observed, as protocol completion time is reduced [45], which may have implications when comparing inter limb measures, particularly in the timed hop tests (duration: ~ 1.6–2 s). The timer starts when the athlete’s heel leaves the ground at the beginning of the test and stops when the athlete completes the 6-m distance; thus, clinicians encounter 4 potential sources of error (1) heel raise; (2) hit start; (3) visually observe the athlete complete the 6 m distance; and (4) hit stop. There will also be normal variation (typical error) in the performance of the athlete. Cumulatively, these factors suggest that caution should be applied when using this test (and in particular a stopwatch) to determine an athlete’s readiness to return to sport, as there may be potential for misclassification due to the inherent range of error sources present in the test.

When deciding testing criteria, clinicians should be cognizant of how they can determine if changes in performance over time (i.e., through rehabilitation) are ‘real’, accounting for the typical measurement error. Importantly, longitudinal tracking during rehabilitation is needed to create a trajectory and allow clinicians to make more informed decisions. In the available literature, the error shown for all 4 hop tests appears acceptable [38]. Previous data indicate that the SHD displays the lowest percentage change required to detect meaningful change beyond typical error (8.09%), with the highest values reported for the T6H (12.96%) [38]. Practically, this means that larger performance changes are required in the execution of the timed hop vs. the other hop tests (and in particular the SHD) to be confident of a meaningful improvement. Target scores for individual athletes (for both hop distance and symmetry) may also be required that consider their specific measurement error, and level of performance, to determine if changes observed in response to targeted rehabilitation are meaningful.

Finally, athlete familiarization is also an important consideration which is often not applied in clinical practice. Reid et al. [41] showed that although LSI was consistent over the first 3 tests, absolute hop distance was not, showing improvements over tests 1 and 2. This indicates that a learning effect which needs to be acknowledged to ensure the athlete’s maximum performance is measured. In addition, the previous data suggest that hop distance (relative to body height) is a predictor for re-injury, alongside LSI in the THD [33]. Therefore, adequate exposure to testing protocols prior to data collection is essential, and relative hop scores need to be considered to ensure that a minimum level of performance is obtained.

### Relationships with Strength

An understanding of the contributing factors that underpin successful hop performance can help clinicians identify potential deficiencies. Asymmetries during the hop for distance tests have been associated with deficits in strength in the involved limb [46–49], and lower knee joint moments and power [50, 51]. However, it is important to recognize that correlation does not equal causation, and often, the strength of these relationships is low to moderate [52, 53], suggesting that other factors are present. Peak torque and rate of torque development have been shown as predictive factors for both single (*R*^2^ = 60.9%) and triple hop for distance (*R*^2^ = 61.8%) [54, 55]. However, maximal rebound hopping requiring multiple contacts appears to be distinct from a single hop and stick task, as reactive strength index (jump height/ground contact time) was included in the regression model for the THD and not the SHD [54].

The CHD and THD share similar qualities or are co-linear, with strong correlations between the two tests (*r* = 0.76) [56]. This might be expected, as the protocol for triple and cross-over hop is inherently similar (Fig. 1), the main difference being that the cross-over hop requires a 15 cm medial–lateral deviation across a tape measure. However, the literature indicates that they may be measuring slightly different constructs. Regression analysis completed for all 4 hop tests showed that asymmetry of strength was a predictive factor for asymmetry in the SHD and THD, but not the CHD and T6H [47]. When interpreting strength data and associations with function, clinicians should consider that only peak knee extensor torque was measured which may not reflect the additional strength qualities that underpin functional performance (reactive, eccentric, rate of force development, frontal and transverse plane control etc.) [57], as well strength in different anatomical locations. For example, hip external rotation strength has been shown to independently predict re-injury risk [58], as well as hop distance deficits in ACLr patients, which are observed throughout rehabilitation [59], yet has received little attention in RTS protocols, or by way of comparisons between hop tests. Cumulatively, the data indicate that performance on different hop tests may provide specific information. For example, higher LSI on a SHD, with lower values in the triple hop, may be indicative of good strength and rate of torque development, but limitations in the ability of the limb to generate sufficient breaking forces, and transmit these into the propulsive phase (reactive strength).

### Relationships with Subjective Function

There may also be a psychological component that accounts for some of the deficits observed during hop tests [53]. Previous research has indicated the CHD shows the strongest relationship with self-reported knee function [60, 61]. This may be due to the greater medio lateral force application during the cross-over deviation which is associated with the injury mechanism [62, 63], resulting in lower confidence levels when an athlete is asked to perform maximally on the involved limb. However, other research has shown that the CHD is the only hop test which is not associated with self-reported knee function [64]. The T6H also appears to have strong associations with perceptions of lower limb performance capacity [49, 60, 61, 64]. For this test, it is more difficult to determine these associations. Perhaps, the distance covered and number of repetitions at high speed may create a more cautious approach from the athlete. In addition, there is also evidence that psychological factors may contribute to SHD and THD performance [60, 64, 65], with Muller et al. [32] demonstrating the SHD to be the strongest predictor of self-reported knee function 1 year after surgery when all 4 tests were used. Cumulatively, relationships with self-reported function would appear to be equivocal, with variation seen across the range of studies.

### Temporal Evaluation (Which Tests Should We Use to Detect Change Over Time?)

**Fig. 3:**
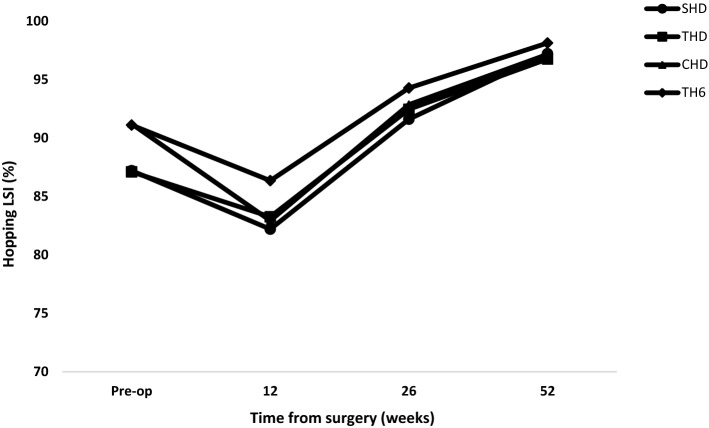
Changing limb symmetry index as a percentage over time from surgery. To avoid rehabiltation protocol bias, only studies that measured all 4 hops longitudinally were selected. Pre-op [66], 12 weeks [38, 63], 26 weeks [38, 63, 66], and 52 weeks [63, 66] (Reid et al. [38] data were taken at 16 and 22 weeks, and have been included as part of the analysis at 12 and 26 weeks, respectively). *SHD* single hop for distance, *THD* triple hop for distance, *CHD* cross-over hop for distance, *TH6* 6 m timed hop, *LSI* limb symmetry index

While the hop tests have often been used as a ‘discharge’ protocol, it is also advised that clinicians monitor function during rehabilitation. Significant changes over time in the injured versus the uninjured limb have been observed for all 4 hop tests [41]; however, a trend for an earlier return to limb symmetry appears to exist for the T6H relative to the other hop tests (Fig. 3) [28, 66–69]. For example, the T6H was the only hop test which was not different between ACL-reconstructed athletes and healthy-matched controls at the time of RTS during a modified NFL combine test [68] and has been unable to differentiate between patient success when monitoring RTS outcomes [32]. These findings might be explained by Hopper et al. [70], who reported that the early normalization shown in the T6H, in comparison with the CHD and single leg vertical jump, may be due to the relatively low demands of the activity. The goal of the task is to hop as fast as you can; thus, athletes tend to adopt more frequent and shorter steps. Consequently, the mechanical joint loads and breaking forces that are required on each ground contact are likely to be lower (although to date no studies have tested this). In addition, the requirement for maximal, rapid deceleration is lower by removing the stop requirement, whereas the other 3 hop tests all require the ability to stick and hold the final hop landing. Comparatively, the SHD appears in a number of studies to have marginally lower values at each time point measured compared to the other three hop tests (Fig. 3), suggesting that it may be a more sensitive temporal measure to differentiate between the previously injured and non-injured limb as well as improvement in overall functional symmetry over time [28, 41, 71]. Strikingly, it can be seen that knee function as measured by each of the hop tests does not appear to plateau over the testing time points commonly used in the ACLr literature (Fig. 3). Although LSI values are within the acceptable range (> 90%) at 52 weeks post-surgery, the data indicate that even at this late stage, the subject’s recovery is still on an upward trajectory. A further assessment time point may have shown diminishing returns from rehabilitation, and perhaps greater readiness to RTS and highlights the potential need for a strategy that looks to record individual progress over time, so the decision about RTS can be based on longitudinal progress, rather than a one-off test.

### Do We Need 4 Hop Tests?

The utility of a test to correctly differentiate an abnormality when one is present (sensitivity), is essential for a clinician to be able to determine whether a genuine deficit is present. Individually, the hop tests show poor sensitivity in their ability to identify deficits (using an LSI threshold of > 85%), with values ranging from 44 to 58% [38, 60, 72]. Even though utilization of 3 or 4 hop tests as the ‘functional’ component in RTS assessment is common [3, 15, 22, 25–27], the ability to observe an abnormality in an ACLr patient using all 4 tests as a ‘battery’ appears to be no greater than just using 2; however, more than one hop test may be required to increase their sensitivity (50% vs. 62% for 1 and 2 tests, respectively) [38]. In addition, there were no 2 hop tests that when performed together, showed any greater sensitivity compared to any other combination of 2 tests [38]. Conversely, Wilk et al. [60] reported the T6H to have a sensitivity of only 26% which is markedly lower than those generally found for the other 3 hop tests; although, when used along with the CHD, this combination showed the highest sensitivity [60]. However, the THD was not included in this study making direct comparisons difficult. A more recent study showed the SHD alone demonstrated equivalent sensitivity to any other two combinations of the hop tests [65]. Overall, these data suggest that using all 4 tests simultaneously is likely surplus to requirements in terms of their ability to detect abnormality.

Correlational analysis (which is not without its limitations) also supports the need for a more selective approach in the use of hop testing. The SHD and THD have reported strong associations (*r* = > 0.8) [56, 72, 73], and similar values have been observed between the SHD and T6H (*r* = 0.89) [74]. Only the SHD and CHD have been shown to have a relatively low correlation (*r* = 0.56) [56], perhaps indicating that these two hops may be measuring different constructs. In addition, a recent study reported that although all 4 tests were consistent predictors of return to high level sporting activities, it was the T6H which contributed most to the regression model at 12 months’ post ACLr, with a quarter of the variance explained by this functional test (*R*^2^ = 0.22). At 24 months, a combination of the SHD and T6H predicted almost half of the variance in RTS at the same pre-injury level, with the other hops adding little to the overall model [30].

Cumulatively, the data indicate that the inclusion of all 4 hop tests as an assessment battery may not be necessary to enhance RTS decision making. The inclusion of more tests that measure similar constructs increases the inherent error associated with execution which comes from many sources (athlete fatigue, motivation, tester error etc.). The inclusion of only 2 tests may also provide additional time to test other important constructs to further guide the clinician regarding the function of their athlete’s knee. Further investigation is warranted to determine if an optimal combination of tests exists that provides the clinician with the most insight into the athlete’s state of readiness to return to play.

## Other Unilateral Jump Test Alternatives to Single Leg Hop Protocols

In addition to streamlining the horizontal hop testing, it may be pertinent to consider other jump and hop tests to ensure all relevant physical constructs are being examined as these may load the knee differently and provide broader challenges for the athlete. For example, lower extremity joint contributions during bilateral jumping showed that the contribution of the knee during the propulsive phase of the horizontal jump was only 4%, compared to 24% in the vertical jump, with the ankle predominantly making up the difference [75]. This suggests that the vertical jump may prove to be a better indicator of the functional capabilities of the knee joint and could be considered as a pertinent test to examine performance following ACLr. In addition, lateral, vertical and horizontal hops could be considered distinctly different tasks by virtue of their moderate associations (*r* < 0.64) [76]. By testing the patient in different planes of motion, the clinician can more clearly identify movement deficits that are relevant in the context of their sport, and these can be subsequently developed through targeted training interventions.

### Unilateral Vertical Hop

The goal of this test is to achieve maximum vertical displacement in a unilateral stance and land under control. Excellent reliability has been shown in both healthy (*r* = 0.86) [73] and ACLr patients (*r* < 0.88) [72, 77, 78]. Heightened between limb differences are expected during this test in healthy populations, with 6% of the cohort showing a limb symmetry score of < 85%, compared to 0% in a horizontal hopping task [73], and 11% scoring lower than 90% LSI [72]. However, while this test may display lower specificity, in comparison with horizontal hops, its sensitivity to identify individuals with a history of ACLr is greater, with values of 86% versus 63% in the vertical jump and SHD, respectively [72]. This is supported by several studies that indicate a delay in athletes achieving 90% single leg vertical jump height symmetry when compared to horizontal hopping after ACLr [79, 80]. However, these findings are not universal with vertical hop demonstrating similar progressions to the CHD, but a trend for slower normalization compared to the T6H [70], as well as greater symmetry throughout rehabilitation compared to the T6H and SHD [71]. The observed differences between studies may be indicative of the prescribed rehabilitation protocols that address distinct physical qualities and movement patterns, as well as variations in test methodologies, and support the need to unify testing protocols and for further research to examine the biomechanics and performance constructs of the individual hop tests.

### Unilateral Repeated Vertical Hop/Rebound Test

Another protocol that has been used within the available literature requires subjects to hop as fast and high as possible for a period of 10 s [39] When using jump height as the variable measured, the sensitivity for this test at 54 weeks’ post-surgery was reported as higher than horizontal hopping at 72% vs. only 28%, and 16% for the SHD and THD, respectively. Specificity was also equal in this test when compared to the two horizontal tests (96%), making it an appealing option to detect abnormalities in the injured population. However, although Petschnig et al. [39] reported ICCs above 0.89 for 10 s of hopping, another study using cyclic vertical hopping reported values for the dominant and non-dominant legs of 0.71 and 0.81, respectively [73]. In addition, the same author found specificity to be lower at higher LSI cut offs compared to vertical and horizontal hops, with 39% of healthy subjects demonstrating asymmetry < 90% LSI. This suggests caution must be applied when using higher cut off values of > 90% for this test as it is possible that injured subjects who have a healthy knee may still report an abnormality in this test when one is not present. Recently, single leg drop jumps have been utilized and have shown greater biomechanical deficits for the involved leg 9 months after reconstruction compared to the single leg hop [81]. This suggests it may be a more sensitive measure to abnormal function over time compared to the horizontal hop; however, further reliability studies and sensitivity testing need to be conducted in ACLr populations to confirm its value as an addition to RTS testing protocols.

### Side Hop/Rotational Hops

The protocol for the side hop involves 30 s of medial–lateral displacement, back and forth between 2 markers placed on the floor positioned 40 cm apart. Mediolateral forces at the knee are considered to be high risk during dynamic movements [62, 63] and so an evaluation of performance in this plane of motion may provide useful information. The test has been shown to have good reliability (*r* > 0.85) [72], and heightened sensitivity in comparison with horizontal hopping, with values of 77% in the side hops, although this was lower than the vertical hop which was 87% in ACLr subjects [72]. However, the same author also reported low specificity of 87% when LSI was set at 90%, suggesting that in healthy subjects, 13% would show up as having an abnormal knee when no injury was present, whereas SHD specificity was at 100%. Similarly, Dingenen et al. [82] reported excellent reliability (*r* > 0.9) in a triple lateral hop for distance, and single rotational hop for distance, and also observed a lower percentage of ACL patients passed the 90% LSI threshold for these tests 6 months after surgery compared to SHD and THD. However, it was also reported that in the healthy population only 69% had a limb symmetry threshold in excess of this cut off, compared to 93% in the horizontal hops. Low test specificity is a key consideration for determining optimal time frames for successful RTS. If the tests selected are prone to identifying an abnormality when there is not one (false-positive), we are potentially withholding an otherwise healthy athlete from returning to sport. This is an important consideration, particularly for the longer term. Thomee et al. [79] reported that only about 50% and 60% of patients had passed the side hop and countermovement jump, respectively, compared to approximately 85% for the single hop at 2 years post reconstruction using a 90% LSI cut off. This indicates a need to reduce the LSI percentage for these tests to ensure greater accuracy of decisions; however, reducing the target LSI percentage value for these tests will in turn reduce the test’s sensitivity. Furthermore, rehabilitation programs may wish to consider including protocols to address these deficits by targeting factors which improve mediolateral force production.

## Can We Use the Contralateral Limb to Guide Performance?

Recent concerns have been raised regarding the use of the uninjured limb as an index measurement during rehabilitation. It is possible that as a result of reduced loading, a progressive detraining effect may result during the period immediately after the injury, and through early rehabilitation. A number of studies have demonstrated reduced absolute distance deficits in both the involved and uninvolved limb of ACLr patients in comparison with healthy-matched controls or preoperative values [26, 83] for up to 24 month post-surgery [84]. Wren et al. [83] observed that although a number of ACLr patients demonstrated limb symmetry, they hopped shorter distances on the uninjured leg compared to asymmetric patients, and a healthy-matched control group. The authors suggest that this may be as a result of deconditioning, fear or lack of motivation, but it also raises the concern that athletes, consciously or subconsciously, may be able to manipulate test performance to expedite their return to play. A practical strategy (in the absence of pre-injury data) that may be implemented to avoid these issues requires conducting assessments on the contralateral limb preoperatively. This would direct the focus during rehabilitation to not only matching LSI, but will also provide an aim of achieving their pre-injury capacity in the contralateral limb. Wellsandt et al. [85] tested the non-injured limb preoperatively and at the point of return to play. They reported that only 29% of patients met hop distance criteria (90% LSI) when using preoperative distance as the comparative measurement, versus 57% when using the non-injured limb post-operative performance as the index measurement. Importantly, when using the preoperative hop distance with the non-injured leg as the reference for symmetry, the data showed greater ability to predict a second ACL injury. Therefore, obtaining data for the contralateral limb as soon as possible after the injury (or surgery if no pre-op values were obtained), and reporting symmetry and relative hop distance performance trajectory on each limb in the later stages of rehabilitation in a performance context as well as just the LSI may give the clinician a more accurate benchmark and estimation of the athlete’s state of readiness for RTS.

### The Importance of Assessing Movement Quality

Although performance outcomes (hop distance/time) may suggest that acceptable symmetry between limbs has been achieved, these measures do not take into consideration information about how the task is executed in terms of movement quality. Quantification of performance outcome measures alone may not be enough. Paterno et al. [58] demonstrated that lower extremity biomechanics during a vertical landing may be predictive of ACL re-injury; specifically, an increase in knee valgus, and greater asymmetry in internal knee extensor moments at initial contact. When tested at the point of return to sports, differences in loading have been observed in the ACL injured limb [18, 50, 86], which may persist for up to 7 years after surgery [87]. Xergia et al. [88] measured joint angles and moments at the hip, knee, and ankle in a group of ACLr patients during a single hop for distance and reported no relationship between the LSI for the variables, when compared to asymmetries in distance hopped, 6–9 months after surgery. Similarly, although LSI single hop scores of > 90% were achieved in patients after ACLr, reductions in peak knee flexion were evident on the involved limb, indicating a compensatory strategy [89]. This either implies that movement quality might appear to progress at a different rate to performance measurements, or that using current LSI guidelines derived from hop distance measured are not suitable guidelines to determine movement quality. Supporting this, Wren et al. [83] showed that the involved knee at ~ 6 month post-surgery displayed reduced knee flexion at initial contact, peak knee flexion, and knee flexion excursion, even though the mean LSI hop distance was 92%. Interestingly, patients who were more asymmetric tended to offload more towards the ankle and symmetric patients towards the hip, suggesting that symmetric patients passed the hop test by developing a more successful compensation strategy in which the load is distributed towards the larger musculature involved in knee joint stabilization. These data indicate that utilizing hop distance alone may not give a full picture of the status of the athlete’s knee function at the time of return, and suggest that while assessing performance during the test (attempting to maximize hop distance), other factors relating to neuromuscular control should also be examined and form part of the RTS decision-making process.

In spite of these findings, it should also be considered that the hop tests were originally developed and widely adopted due to their practical utility and time efficiency. Measurement of biomechanics during these tests has not been commonplace, likely due to the expensive equipment and labor-intensive analysis procedures. The use of video cameras positioned in the frontal and sagittal plane has been shown to provide a practically viable option for clinicians, whereby the data captured can be exported and analyzed using freely available software [89]. Recent improvements in wearable technology also provide more feasible options for clinicians which allow them to make more informed and objective decisions. For example, inertial sensors can easily attach to the thigh and shank, to measure knee joint kinematics and have been shown to provide accurate and reliable measures of angular velocity associated with deficits in knee power in ACL injured athletes [90]. Peebles et al. [86] used a “relatively inexpensive” single sensor force insole that can be inserted into the patient’s shoe to provide real time feedback on impact force, loading rate, and impulse with good reliability [91]. These variables displayed enough sensitivity to confirm limb asymmetries in loading between the injured and uninjured limb. Further research is warranted using a range of wearable technologies to identify what is considered ‘normal’ for a range of pertinent variables provided from such devices in both athletes with a history of ACLr and matched controls. However, clinicians are encouraged to thoroughly research the product and, where possible, perform the required analysis to determine their validity and reliability.

## Conclusion

The current evidence indicates a lack of consistency in the ability of hop testing used as a measure to assess function following ACLr to predict successful outcomes following rehabilitation, either in terms of returning to previous performance levels, or identifying those at a greater risk of re-injury. In addition, the current practice of using all 4 tests to inform decision making appears to be unnecessary, with the evidence, suggesting that the single hop and triple hop for distance appear to give the clinician sufficient information, as well as providing an assessment of distinctly different physical constructs. Eliminating the need to perform all 4 hop tests allows the inclusion of other ‘hops’ that may offer different insights into the functional status of the knee, and the athlete’s readiness to return to sport. Moving forwards, other factors relating to neuromuscular/movement control (as an additive to just hop distance/time) should be examined and form part of the RTS decision-making process. Finally, the use of pre-injury hop distance on the contralateral leg as an index measure is recommended, not only as a gauge for reducing re-injury risk, but also as a target to help the athlete to reach the previous performance capacity.
